# Efficacy and tolerability of cryosurgery followed by a topical agent for multiple actinic keratosis: A systematic review and network meta-analysis

**DOI:** 10.1016/j.jdin.2025.10.010

**Published:** 2025-10-25

**Authors:** Mercedes Sanchez-Diaz, Juan Rivera-Quispe, Leandro Huayanay, Erika Romero-Sandoval, Frine Samalvides

**Affiliations:** a Facultad de Medicina Alberto Hurtado, Universidad Peruana Cayetano Heredia, Lima, Peru; b Departamento de Enfermedades Infecciosas, Tropicales y Dermatologicas, Hospital Cayetano Heredia, Lima, Peru

*To the Editor:* Actinic keratosis (AK) is a common skin condition in older adults, characterized by atypical proliferation of keratinocytes due to chronic UV radiation exposure. Its estimated prevalence is approximately 19%.^1^ For patients with multiple lesions, combination therapies—particularly cryosurgery followed by a topical agent—are widely used and recommended.^2^^,^^3^ A conventional meta-analysis by Heppt et al suggested improved outcomes with combination therapy.^4^ However, the comparative efficacy of specific topical adjuvants remains unclear.

To address this gap, we conducted a systematic review and network meta-analysis of randomized controlled trials (RCTs) evaluating cryosurgery followed by a topical agent for AK. This approach allowed both direct and indirect comparisons across interventions and allowed for treatment ranking. Eligible RCTs enrolled patients with ≥2 lesions and had a treatment duration of ≤24 weeks. Full methodology is available in (Supplementary Appendix 1, available via Mendeley at https://doi.org/10.17632/jnff66r7c5.1).

We identified 8 RCTs (*n* = 1478 patients) evaluating 6 agents: imiquimod 3.75%, imiquimod 5%, ingenol mebutate 0.05%, ingenol mebutate 0.015%, diclofenac 3%, and 5-fluorouracil (5-FU) 0.5% (Table I), Supplementary Appendix 1, available via Mendeley at https://doi.org/10.17632/jnff66r7c5.1. Two studies had moderate and 4 had high risk of bias (Supplementary Appendix 2, available via Mendeley at https://doi.org/10.17632/jnff66r7c5.1). Three used intraindividual (split-body) designs. Due to the limited number of studies, a funnel plot was not generated.

**Table I:** Characteristics of included RCTs

Study	Study design	Arm	Cryo technique	Topical regimen	Participants	Mean age (SD)	Male (%)	Mean AK lesion (SD)	Body part	Type of AK lesion	Phototype type I/II	D1	D2	D3	D4	D5	Risk of bias
Jorizzo (2010)	Randomized interindividual, double-blind study	Cryosurgery/Imiq 3.75%	Not described	Imiq 3.75% daily, 2 wk on, 2 wk off, and 2 wk on	I: 126	I: 66 (9.5)	86.0%	I: 16.1 (5.8)	Face	Nonhypertrophic	70.80%	Y	Y	Y	Y	Y	Low
		Cryosurgery/Placebo			C: 121	C: 67.3 (10)	87.6%	C: 15.8 (5.8)									
Rigel (2010)	Randomized intraindividual, open-label, assessor-blinded study	Cryosurgery/Imiq 5%	Not described	Imiq 5% 3 times per wk for 4 wk	I&C: 27	I&C: 67.8 (9.2)	96.3%	I: 8.7 (2.2)	Face/scalp	Nonhypertrophic	NR	U	N	Y	Y	Y	High
		Cryosurgery alone						C: 8.5 (2.3)									
Tan (2007)	Randomized, interindividual, double-blind study	Cryosurgery/Imiq 5%	3-5 s freeze cycle	Imiq 5% 2 times per wk for 8 wk	I: 33	I: 71 (8)	87.9%	I: 8.5 (3.2)	Face/scalp	Any AK lesion	NR	Y	U	Y	Y	U	Moderate
		Cryosurgery/Placebo			C: 32	C: 69.4 (7.6)	87.5%	C: 8.1 (3.6)									
Goldenberg (2013)	Randomized. intraindividual, assessor-blinded study	Cryosurgery/Imq 3.75%	Two 5-s freeze cycle with 5-s rest interval	Imiq 3.75%, 2 wk on, 2 wk off, and 2 wk on	I&C: 20	I&C: 73.4 (NR)	85%	I: 6.0 (NR)	Dorsal hand/forearm	Hypertrophic	NR	U	N	N	N	U	High
		Cryosurgery alone						C: 5.8 (NR)									
Hashim (2014)	Randomized, intraindividual, assessor-blinded study	Cryosurgery/IngMeb 0.05%	Two 5-s freeze cycle with 5-s rest interval	IngMeb 0.05% daily for 2 consecutive days∗	I&C: 16	I&C: 67.0 (9.8)	93.8%	I: 5.4 (NR)	Dorsal hand	Hypertrophic	NR	Y	N	Y	Y	Y	High
		Cryosurgery alone						C: 4.5 (NR)									
Berlin (2008)	Randomized, interindividual, open-label study	Cryosurgery/Dic 3%	Single 4-10 s freeze cycle	Dic 3% twice daily for 90 d	I: 368	I&C: 70.0 (NR)	76%	I: 8.9 (NR)	Face/scalp/hands	Any AK lesion	90%	N	N	N	N	U	High
		Cryosurgery alone			C: 346			C: 8.2 (NR)									
Beman (2014)	Randomized interindividual, double-blind study	Cryosurgery/IngMeb 0.015%	Not standardized	IngMeb 0.015% for 3 consecutive days∗	I: 167	I: 66.7 (NR)	82.6%	I: 5.7 (NR)	Face/scalp	Nonhypertrophic	63.20%	Y	Y	Y	Y	Y	Low
		Cryosurgery/Placebo			C: 162	C: 67.4 (NR)	82.1%	C: 5.8 (NR)									
Hoover (2014)	Randomized interindividual, assessor blinded	Cryosurgery/5-FU 0.5%	Not described	5-FU 0.5% daily for 1 wk†	I: 30	I: 67.5 (NR)	90.0%	I: 12.0 (NR)	Face/scalp	Any AK lesion	NR	Y	Y	Y	Y	U	Moderate
		Cryosurgery Placebo			C: 30	C: 66.5 (NR)	93.0%	C: 12.0 (NR)									

Mod as an adjunct to cryotherapy for actinic keratoses. J Cutan Med Surg. 2007;11(6):195-201. ClinicalTrials.gov. NCT00110682. Study of Imiquimod 5% Cream in Addition to Cryotherapy in the Management of Actinic Keratoses. Berman B, Goldenberg G, Hanke CW, et al. Efficacy and safety of ingenol mebutate 0.015% gel 3 weeks after cryosurgery of actinic keratosis: 11-week results. J Drugs Dermatol. 2014;13(2):154-160. ClinicalTrials.gov. NCT01541553. A Sequential Treatment Regimen of Cryotherapy and Picato for the Treatment of Actinic Keratosis on the Face and Scalp. Hoover WD, Jorizzo JL, Clark AR, Feldman SR, Holbrook J, Huang KE. Efficacy of cryosurgery and 5- fluorouracil cream 0.5% combination therapy for the treatment of actinic keratosis. Cutis. 2014;94(5):255-259. Goldenberg G, Linkner RV, Singer G, Frankel A. An Investigator-initiated Study to Assess the Safety and Efficacy of Imiquimod 3.75% Cream When Used After Cryotherapy in the Treatment of Hypertrophic Actinic Keratoses on Dorsal Hands and Forearms. J Clin Aesthet Dermatol. 2013;6(2):36-43. ClinicalTrials.gov. NCT00110682. Imiquimod 3.75% Cream in Combination With Cryotherapy in the Treatment of Hypertrophic Actinic Keratoses. Hashim PW, Nia JK, Singer S, Goldenberg G. An Investigator-initiated Study to Assess the Safety and Efficacy of Ingenol Mebutate 0.05% Gel When Used After Cryosurgery in the Treatment of Hypertrophic Actinic Keratosis on Dorsal Hands. J Clin Aesthet Dermatol. 2016;9(7):16-22. ClinicalTrials.gov. NCT02251652. Safety and Efficacy of Ingenol Mebutate 0.05% Gel When Used After Cryotherapy in the Hypertrophic Actinic Keratoses. Berlin JM, Rigel DS. Diclofenac sodium 3% gel in the treatment of actinic keratoses postcryosurgery. J Drugs Dermatol. 2008;7(7):669-673.

*5-FU*, 5-Fluorouracil; *C*, comparator group; *Cryo*, cryosurgery; *D1*, domain 1 (randomization process); *D2*, domain 2 (deviations from the intended interventions); *D3*, domain 3 (missing outcome data); *D4*, domain 4 (measurement of the outcome); *D5*, domain 5 (selection of the reported result); *Dic*, diclofenac; *I&C*, interindividual studies; *I*, intervention group; *Imq*, imiquimod; *IngMeb*, ingenol mebutate; *N*, no; *NR*, not reported; *P*, placebo; *RCT*, randomized controlled trial; *U*, unknown; *Y*, yes.

∗ Ingenol Mebutate was withdrawn from the market in US and European Union due to safety concerns in 2020.

† Off-label use for filed therapy.

We performed separate network meta-analyses for complete clearance (100% lesion reduction) and partial clearance (≥75% reduction). Complete clearance was assessed in 7 RCTs. Four therapies showed statistically significant benefit over cryosurgery alone: cryosurgery/5-FU 0.5% (risk ratio [RR] 3.00; 95% CI 1.09-8.25), cryosurgery/diclofenac 3% (RR 1.82; 95% CI 1.37-2.25), cryosurgery/imiquimod 3.75% (RR 9.12; 95% CI 3.36-24.79), and cryosurgery/ingenol mebutate 0.05% (RR 19.00; 95% CI 1.20-300.42) (Fig 1).

**Fig 1:**
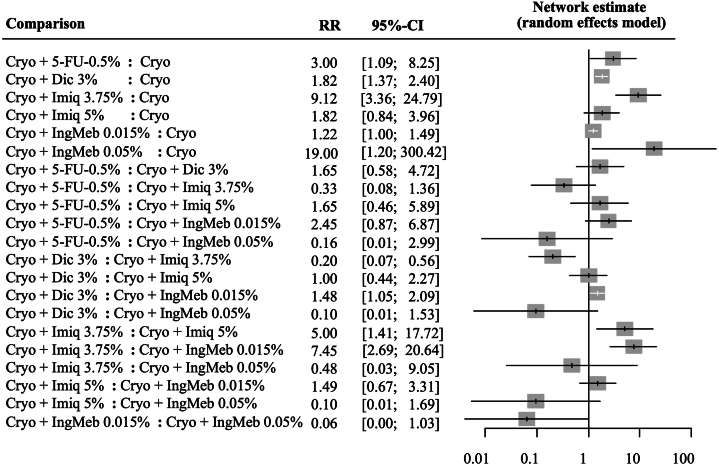
Forest plot of network meta-analysis for outcome complete clearance (without sensitivity analysis). *Cryo*, Cryosurgery; *Imiq*, imiquimod; *IngMeb*, ingenol mebutate; *Dic*, diclofenac; *5-FU*, 5-fluorouracil; *RR*, risk ratio.

Treatment ranking based on P-scores showed that cryosurgery/ingenol mebutate 0.05% was most likely to be the most effective (P-score 0.96), followed by cryosurgery/5-FU 0.5% (P-score 0.69) and cryosurgery/imiquimod 3.75% (P-score 0.68). After excluding high-risk studies, only cryosurgery/5-FU 0.5% (P-score 0.79) and cryosurgery/imiquimod 3.75% (P-score 0.76) consistently ranked highest (Supplementary Appendix 3, available via Mendeley at https://doi.org/10.17632/jnff66r7c5.1). Of note, ingenol mebutate has since been withdrawn in both the United States and European Union due to concerns about skin cancer risk. Certainty of evidence was rated very low using the CINeMA (Confidence In Network Meta-Analysis) tool, primarily due to the small number of studies and limited indirect evidence.

For partial clearance, 3 RCTs assessed combination therapies, and ranking was as follows: ingenol mebutate 0.05% (P-score 0.9), followed by 5-FU 0.5% (P-score 0.68), and ingenol mebutate 0.015% (P-score 0.4), though certainty remained very low (Supplementary Appendix 4, available via Mendeley at https://doi.org/10.17632/jnff66r7c5.1). However, the certainty of evidence for both complete and partial clearance outcomes, as assessed by the CINeMA tool, was rated very low, primarily due to the small number of studies and limited indirect evidence.

vAs for other outcomes, combination therapy also resulted in a greater reduction in total lesion count (Supplementary Appendix 6, available via Mendeley at https://doi.org/10.17632/jnff66r7c5.1). Adverse events, local skin reactions, and patient satisfaction were inconsistently reported, and thus no conclusions were drawn (Supplementary Appendices 6-8, available via Mendeley at https://doi.org/10.17632/jnff66r7c5.1).

Limitations include the small number of trials, limited network connectivity, heterogeneous cryosurgery protocols, varying AK subtypes, and moderate to high risk of bias in several included studies. Notably, the study assessing 5-FU 0.5% (Hoover 2014, see Table I) used an off-label dose.

Despite the low overall certainty of evidence, cryosurgery plus either 5-FU 0.5% or imiquimod 3.75% appear to be the most adjuvant strategies for AK. Well-designed, adequately powered RCTs with standardized outcomes are needed.^5^

## Conflicts of interest

None disclosed.
